# Peripheral Ulcerative Keratitis Associated With Large Vessel Vasculitis

**DOI:** 10.7759/cureus.15767

**Published:** 2021-06-20

**Authors:** Shun Uchida, Yuki Kaji, Mutushito Ui, Hirotoshi Kawashima, Tomohiko Usui, Yoshiyuki Ohira

**Affiliations:** 1 General Medicine, International University of Health and Welfare Narita Hospital, Chiba, JPN; 2 Allergy and Rheumatology, International University of Health and Welfare Narita Hospital, Chiba, JPN; 3 Ophthalmology, International University of Health and Welfare Narita Hospital, Chiba, JPN

**Keywords:** peripheral ulcerative keratitis, giant cell arteritis, large vessel vasculitis, 18fdg-pet, episcleritis

## Abstract

Peripheral ulcerative keratitis (PUK) is a non-infectious ulcer at the peripheral corneal stroma. Autoimmune diseases can cause PUK, but PUK caused by large vessel vasculitis (LVV) has rarely been reported. We report the case of a 71-year-old woman with complaints of low-grade fever and left eye pain. Ophthalmologic examination revealed PUK in the left eye, and we diagnosed LVV by ^18^F-fluorodeoxyglucose-positron emission tomography (FDG-PET) findings. The patient was treated with topical betamethasone eye drops for PUK and oral prednisolone for LVV. This case suggests that LVV can cause PUK.

## Introduction

Peripheral ulcerative keratitis (PUK) is a non-infectious ulcer at the peripheral corneal stroma. PUK causes eye pain, ocular redness, tearing, photophobia, corneal opacity, and vision loss [1]. Autoimmune diseases are the most common cause of PUK, with rheumatoid arthritis accounting for 34%-42%; systemic lupus erythematosus, relapsing polychondritis, antineutrophil cytoplasmic antibody-associated vasculitis, and polyarteritis nodosa have also been reported [2]. PUK caused by large vessel vasculitis (LVV) has rarely been reported. According to the nomenclature reported by International Chapel Hill Consensus Conference, LVV is a vasculitis that affects large arteries and includes giant cell arteritis (GCA) and Takayasu arteritis (TKA) as major variants [3]. The frequency of PUK associated with GCA is unknown, and only one case has been reported [4]. There are no reports about PUK associated with TKA. Other causes of PUK include local eye infections and mucosal skin disorders [5]. Depending on the primary disease, systemic steroids are the mainstay of treatment for PUK [1]. We encountered a case of PUK associated with LVV and report the usefulness of 18F-fluorodeoxyglucose-positron emission tomography (FDG-PET) for the diagnosis of LVV.

## Case presentation

**Figure 1:**
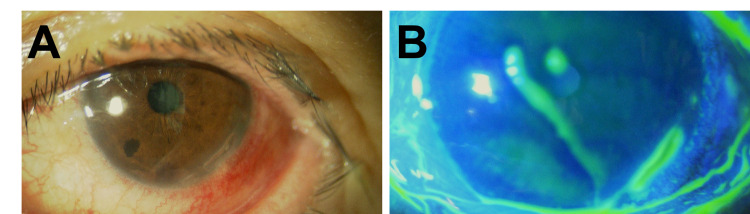
Clinical photograph of the affected eye prior to the therapy. Slit-lamp photograph (A) and staining with fluorescein (B) showed an arcuate-shaped peripheral corneal stroma, which is specific for PUK. PUK, peripheral ulcerative keratitis

A 71-year-old woman was hospitalized for low-grade fever for two weeks, malaise, back pain for one week, and pain and hyperemia in her left eye for one day. There were no other symptoms such as headache, neck pain, or joint pain. Her medical history was notable for hypertension treated with cilnidipine and candesartan cilexetil. There was nothing special to note on familial history. Her body temperature was 37.9°C. Ophthalmologic examination revealed an arcuate-shaped peripheral corneal stroma with episcleritis in her left eye, which suggested PUK (Figure 1).

**Figure 2:**
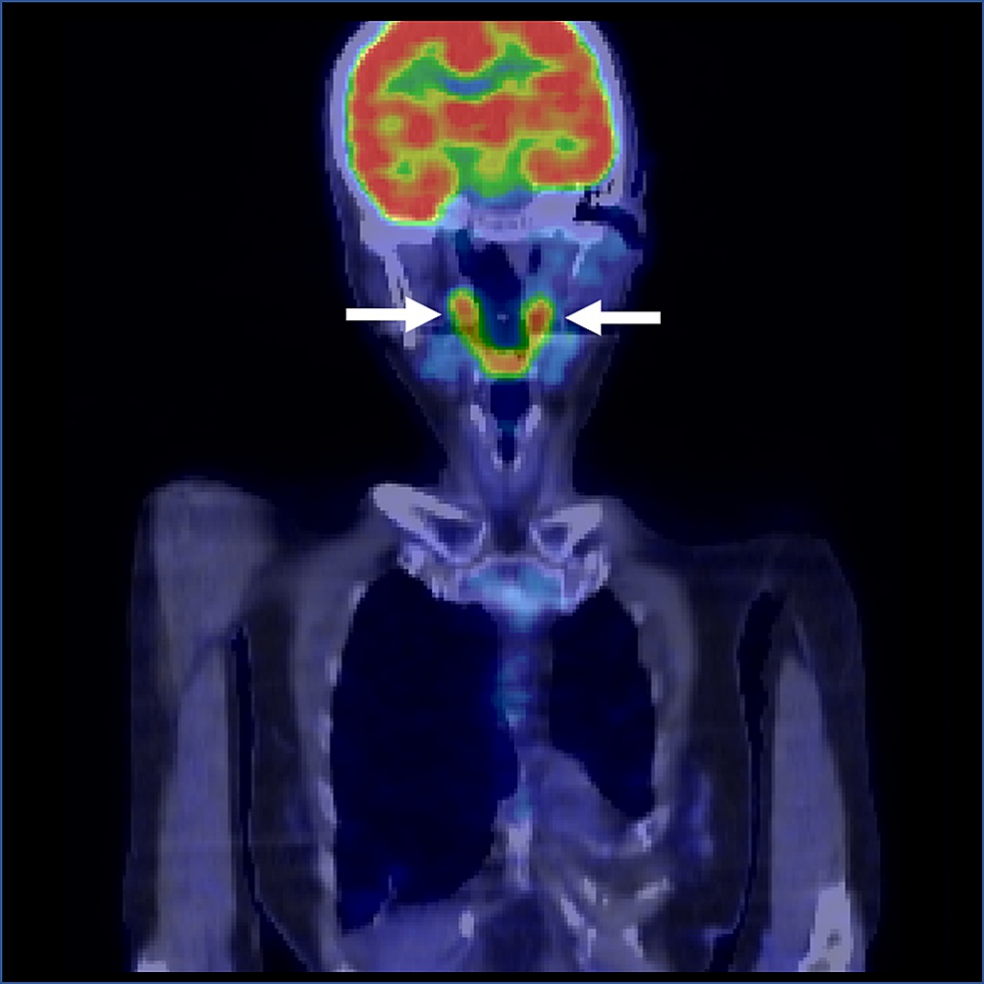
FDG-PET-CT. FDG-PET-CT revealed accumulations in the carotid arteries and pulmonary arteries bilaterally FDG-PET-CT, 18F-fluorodeoxyglucose-positron emission tomography-computed tomography

There were no other significant physical findings. Laboratory tests were notable for white blood cell count, 12.35 × 109/L, erythrocyte sedimentation rate, 117 mm/h, and C-reactive protein, 16.47 mg/dL. Other specific tests were negative, including rheumatoid factor, anti-cyclic citrullinated peptide antibody, anti-nuclear antibodies, and anti-neutrophil cytoplasmic antibody. Magnetic resonance imaging of the lumbar spine revealed osteophyte formation and edema, which suggested sacroiliac arthritis. Whole-body contrast CT and ultrasound to temporal arteries and carotid arteries did not reveal any significant findings. To further investigate the cause of fever and inflammation, FDG-PET was performed and showed accumulations in the carotid arteries and pulmonary arteries bilaterally (Figure 2). There were no findings in the sacroiliac joints or the temporal arteries. Another FDG-PET finding was accumulation in the thyroids diffusely. Thyroiditis was suspected but no therapeutic intervention was performed because her thyroid function was normal.

**Figure 3:**
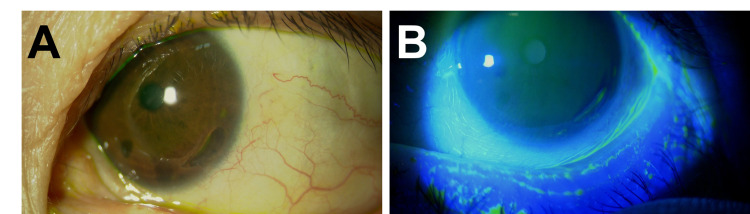
Clinical photograph of the affected eye after the therapy. Slit-lamp photograph (A) and staining with fluorescein (B) did not show PUK findings PUK, peripheral ulcerative keratitis

Based on the FDG-PET findings, she was diagnosed with LVV in the Rheumatology Department. Giant cell arteritis (GCA) was suspected based on an onset age of over 50 years and an increased erythrocyte sedimentation rate, but a pathological study could not be performed because there was no adequate artery for biopsy. According to the rheumatologist’s evaluation, her back pain and sacroiliac arthritis could be due to polymyalgia rheumatica (PMR), a common complication of GCA [6]. Topical betamethasone eye drops (four times/day) were started, and the symptoms of her left eye improved shortly. After a diagnosis of LVV and PMR, 30 mg/day of oral prednisolone (0.6 mg/kg body weight) was started. This prednisolone dosage was determined by the rheumatologist according to the Japanese guideline of 0.5-1.0 mg/kg [7]. After a few days, her fever and back pain improved. After three weeks, the inflammation improved, and the PUK findings completely disappeared (Figure 3). The dose of oral prednisolone was tapered down, and the symptoms did not recur.

## Discussion

This case suggests that LVV can cause PUK and that FDG-PET is useful for the diagnosis of LVV. In the present case, the complication of PUK with GCA was confirmed. In the pathogenesis of PUK, immune complexes may be deposited in the periphery of the cornea and cause inflammation [1]. In the present case, there were also episcleritis findings. Episcleritis associated with GCA or TKA has been reported previously [8-9]. It is speculated that PUK associated with LVV arises through pathogenesis similar to that of episcleritis, which has already been reported as a complication.

The usefulness of FDG-PET in the diagnosis of LVV has already been reported [10]. In the present case, the possibility of GCA was mainly considered because of the patient's age. Criteria for classification of GCA by the American College of Rheumatology [11] include the age of onset >50 years, temporal artery findings, inflammatory findings, and vasculitis findings with giant cells. There are no serologically specific markers for a GCA diagnosis. Temporal artery findings are not always present (15%-73%), and associated symptoms of jaw or tongue claudication are less frequent (20%-48%) [6]. Therefore, cases with no specific findings of GCA, such as the present case, can be challenging to diagnose. The same can be said about TKA owing to the lack of serologically specific markers for TKA. Imaging modalities not always reveal the findings of LVV. For example, according to past reports, systematical color duplex ultrasonography revealed large vessel involvement in 30% of patients with GCA, and CT angiography revealed aortitis in 45%-65% of recently diagnosed GCA patients [12]. In cases of LVV, FDG-PET showed increased uptake of FDG, reflecting vasculitis, with a sensitivity of 90% and a specificity of 98% [10]. Although FDG-PET is useful for the diagnosis and activity assessment of LVV, it is still not standardized because the cut-off setting is controversial. One problem is that uptake of FDG also increases owing to atherosclerosis, which is frequently seen in older age [12]. In the present case, there were only two findings: PUK and systemic inflammation. Although there were no specific findings associated with LVV, considering the possibility of autoimmune disease as a cause of PUK, FDG-PET was examined and LVV could be diagnosed.

## Conclusions

This case suggests that LVV can cause PUK.
